# A Comparison of the Outcomes of COVID-19 Vaccinated and Nonvaccinated Patients Admitted to an Intensive Care Unit in a Low-Middle-Income Country

**DOI:** 10.1155/2024/9571132

**Published:** 2024-10-04

**Authors:** Waleed Bin Ghaffar, Muhammad Faisal Khan, Moeed Bin Abdul Ghaffar, Muhammad Sohaib, Asma Rayani, Muhammad Mehmood Alam, Syed Talha Sibtain, Zahra Cheema, Asad Latif

**Affiliations:** ^1^Department of Anaesthesiology, Aga Khan University Hospital, Karachi P. O. Box 3500, Pakistan; ^2^Department of Surgery, Aga Khan University Hospital, Karachi P.O. Box 3500, Pakistan; ^3^Department of Medicine, Aga Khan University Hospital, Karachi P.O. Box 3500, Pakistan

**Keywords:** acute kidney injury, adult respiratory distress syndrome, COVID-19, COVID-19 vaccines, critical care outcomes, healthcare-associated pneumonia, respiratory insufficiency

## Abstract

Patients critically afflicted with coronavirus disease 2019 (COVID-19) often need intensive care unit (ICU) admission, despite comprehensive vaccination campaigns. The challenges faced by healthcare systems in low-middle-income countries, including limited infrastructure and resources, play a pivotal role in shaping the outcomes for these patients. This study aimed to meticulously compare outcomes between COVID-19 vaccinated and nonvaccinated patients admitted to the ICU. In addition, demographic factors and the ICU course influencing mortality were also assessed. A retrospective review of records from the COVID-ICU of Aga Khan University Hospital spanning July 2021–March 2022 included 133 patients. Statistical analyses, encompassing the Mann–Whitney U-test and chi-square/Fisher exact test, discerned quantitative and qualitative differences. Stepwise multivariable logistic regression models with forward selection identified factors associated with hospital mortality. Results revealed comparable cohorts: vaccinated (48.13%) and nonvaccinated (51.87%). Vaccinated individuals, characterized by advanced age and higher Charlson Comorbidity Index, exhibited more critical disease (89.1%; *p* value: 0.06), acute respiratory distress syndrome (96.9%; *p* value: 0.013) and elevated inflammatory markers. Despite these differences, both cohorts exhibited similar overall outcomes. Factors such as decreased PaO2/FiO2 ratio on admission and complications during ICU stay were significantly associated with in-hospital mortality. In conclusion, despite advanced age and increased frailty among vaccinated patients, their mortality rate remained comparable to nonvaccinated counterparts. These findings underscore the pivotal role of vaccination in mitigating severe outcomes within this vulnerable population.

## 1. Introduction

The coronavirus disease 2019 (COVID-19) was recognized as a pandemic in the year 2020. The burden of disease was much greater than expected in both developing and developed countries resulting in adverse economic impact [1]. Patients with COVID-19 have a varied clinical spectrum from asymptomatic to multiorgan dysfunction [2].

COVID-19 is categorized according to its severity into mild, moderate and severe types. Patients requiring intensive care unit (ICU) admission usually suffer from severe–critical disease. The ICU mortality of these patients ranges from 28.3% to 85% [3]. However, the majority of the information is from developed countries. Limited research from Pakistan has shown that COVID-19 has caused unfavourable economic consequences in the health sector [4].

In Pakistan, the vaccination campaign against COVID-19 commenced with the introduction of five vaccines. The vaccines include inactivated (Sinopharm, Sinovac) and viral vector (CanSinoBIO, Sputnik V, AstraZeneca) in February 2021 [5]. Later, mRNA vaccines (Pfizer, Moderna) were also introduced in May and July 2021 [6–8]. Despite the mass vaccination campaign, people still became infected and required ICU admission. The Aga Khan University Hospital (AKUH) is one of the major referral centres in the country. After the COVID-19 pandemic began, the hospital came to the forefront to cater to such patients.

For patients with severe–critical COVID-19 requiring organ support, a dedicated COVID-ICU was formed. A local study demonstrated better outcomes for vaccinated patients, but there were too few patients with critical COVID-19 to draw any conclusions [9]. The purpose of this study was to evaluate the outcome of severe and critical COVID-19 vaccinated patients in comparison with unvaccinated patients in an ICU.

## 2. Materials and Methods

This retrospective case–control study was conducted at the AKUH. All patients admitted to the COVID-ICU of AKUH between 1 July 2021 and 31 March 2022 were reviewed. Our primary objective was to compare the outcome and the length of hospital stay among the COVID-19 vaccinated and nonvaccinated patients admitted to the COVID-ICU. The secondary objective was to evaluate the effect of demographics and the ICU course on the outcome of these patients. COVID-19 was confirmed in all patients using the polymerase chain reaction (PCR) with either a nasal swab for nonintubated patients or a minibronchoalveolar lavage for intubated patients. Patients with negative COVID-19 PCR and incomplete medical records were excluded.

Data were collected after approval from the departmental research committee and exemption from the ethical review committee of the hospital (ERC number 2022-6783-22392). The demographic data, comorbidities, vaccination status and patient characteristics on ICU admission were noted from patient files. The data were used to calculate the Charlson Comorbidity Index (CCI). Based on the severity of the disease, patients were categorized into severe and critical. COVID-19 was classified by the Society of Critical Care Medicine. Severe COVID-19 was defined as clinical signs of pneumonia and either of the following: respiratory distress, respiratory rate > 30 breaths/min and oxygen saturation < 90% room air. Critical COVID-19 was defined as patients with acute respiratory distress syndrome (ARDS)/respiratory failure requiring ventilation, sepsis or septic shock [10]. Pulmonary artery hypertension was assessed in selected patients suspected of having the condition through echocardiography. As per the European Society of Cardiology, a mean pulmonary artery pressure exceeding 20 mmHg was indicative of pulmonary artery hypertension [11].

Clinical examination data were obtained from the ICU progress and nursing sheet. Patient investigations (laboratory, radiology and microbiology) were obtained from the online portal (patient care inquiry). The medication record was also available from the online portal. The mode of ventilation, the number of prone sessions, organ support, complications and outcome were also documented in the ICU progress and nursing sheet. In our institute, one session comprised 16 h of the prone position. All patients were followed until hospital discharge.

The statistical analysis was carried out using Rstudio 4.1.2 (R Foundation for Statistical Computing). The Shapiro–Wilk or Kolmogorov–Smirnov tests were employed to validate the normality assumption of numerical variables such as age, BMI and invasive ventilation days. For descriptive analysis, frequency (%) estimates of categorical variables were computed, while median ± interquartile range (IQR) estimates of numerical variables were estimated based on the non-normal assumption. The purpose of stratification analysis was to control the effect of all qualitative and quantitative variables for vaccinated and nonvaccinated patients as well as hospital mortality and discharge home groups. The Mann–Whitney U-test (for quantitative) and chi-square/Fisher exact test (for qualitative) were used to establish statistically significant differences between vaccinated and nonvaccinated patients, alongside hospital mortality and discharge home groups. Stepwise multivariable logistic regression models were performed with the forward selection method for evaluating the factors associated with vaccination status and hospital mortality. In addition, the Kaplan–Meier method was employed to calculate the overall survival rate of vaccinated and nonvaccinated patients. Furthermore, the Mantel–Cox Log-rank test was used to analyse the differences in survival rates. The threshold for statistical significance was considered at *p* < 0.05.

## 3. Results

During the study period, 140 patients were admitted to the COVID-ICU. After excluding 7 patients due to missing records, unavailability of files or negative COVID-19 PCR, data from 133 severe to critical illness patients were included in this study.

Demographics and characteristics of hospital admission are summarized in Table 1. Patients had a mean age of 60 years. It is estimated that 43.6% of the patients were in the age range of 40–64. The mean CCI was 3. The majority (82.7%) of patients were admitted due to critical COVID-19. ARDS was most prevalent on admission with a mean PaO2/FiO2 ratio of 142. Laboratory parameters including white blood cell count, C-reactive protein, ferritin and D-dimer were also checked. A total of 64 (48.1%) of the admitted patients were vaccinated against COVID-19. The vaccinated cohort comprised significantly older individuals (mean age: 65.5 years; *p* value: < 0.001) with a higher CCI (*p* value: 0.021). These patients also showed more critical COVID-19 (89.1%; *p* value: 0.06), ARDS (96.9%; *p* value: 0.013) on hospital admission and marked elevations of inflammatory markers, such as ferritin.

The demographics and characteristics of hospital admission were also assessed against hospital mortality. Advanced age (65 vs. 48, *p* value: < 0.001), higher CCI (3 vs. 1; *p* value: 0.003), decreased PaO2/FiO2 ratio (125; *p* value: < 0.001), raised white blood cell count (15.4; *p* value: 0.04) and ferritin (1310; *p* value: < 0.001) were significantly associated with hospital mortality. Further details of the remaining variables including septic shock and myocarditis are shown in Table 1.

Table 2 shows the clinical characteristics of the patients, organ support and complications during ICU stay. Invasive ventilatory support was required for 96.2%, and 62.4% of patients were positioned prone with a median of 4 sessions. The nonvaccinated patients required more organ support, including postextubation respiratory support, cardiovascular support (vasopressors and inotropes) and renal replacement therapy. There was a significant association between these variables and hospital mortality. Management was augmented by pharmacological therapies. The usage of remdesivir and tocilizumab was higher in vaccinated patients. The most common complication developed by patients in both cohorts was acute kidney injury, affecting 60.9% of patients. There was a significant association between complications and hospital mortality (*p* value: < 0.001). The subcategories of complications, comparison between vaccinated and nonvaccinated patients and association with hospital mortality can be seen in Table 2. Among the survivors, the ICU duration and days of invasive ventilation were significantly less. There was no difference in ICU/hospital duration, days of invasive ventilation and ICU/hospital mortality between vaccinated and nonvaccinated patients.

As shown in Table 3, a significant difference between vaccinated and nonvaccinated patients among several covariates was found using stepwise logistic regression with forward selection. These include age, male gender, cardiovascular support and pulmonary artery hypertension. The vaccinated patients suffered more from pulmonary artery hypertension. The forward selection for hospital mortality revealed that PaO2/FiO2 on hospital admission and complications during ICU stay were associated with increased hospital mortality. The vaccination status was not protective.

**Figure 1:**
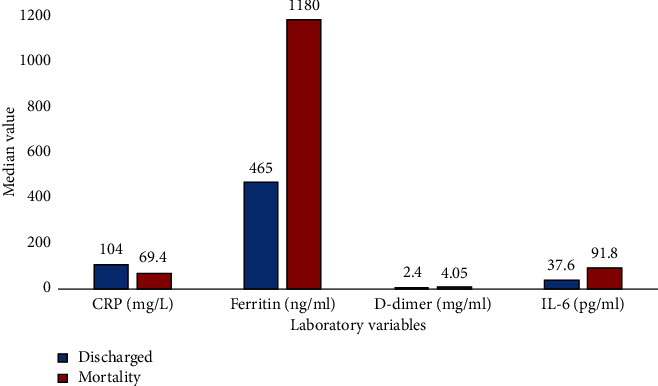
Laboratory parameters on ICU admission in relation to hospital mortality. Abbreviations: CRP, C-reactive protein; IL-6, interleukin 6.

Figure 1 shows the link between elevated laboratory values on ICU admission and mortality. Raised D-dimer, ferritin and interleukin-6 were associated with mortality. C-reactive protein was the sole exception in this case.

**Figure 2:**
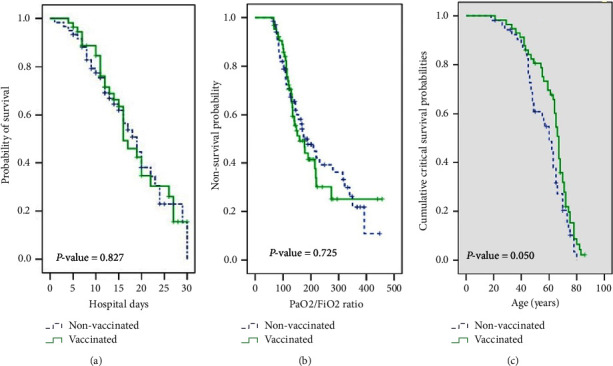
Kaplan–Mier 30-day survival analysis (a), survival probability with the PaO2/FiO2 ratio (b) and survival probability in comparison with age (c) of vaccinated and nonvaccinated patients.

The Kaplan–Meier survival 30-day analysis of both cohorts can be seen in Figure 2(a). The highest mortality rates occurred in the first twenty days after hospitalization, and the outcome of both cohorts was the same.  Figure 2(b) shows the survival probability with the PaO2/FiO2 ratio. The comparison of critical nonsurvival probability in comparison with age can be seen in Figure 2(c). As shown in the figure as the age increases the vaccinated patients had a higher survival probability, but it was not statistically significant (*p* value: 0.05).

## 4. Discussion

The vaccination against COVID-19 has indeed reduced the severity of disease, morbidity and mortality [12–14]. However, whether or not vaccination reduces the occurrence of critical COVID-19 remains unanswered. In addition, low-middle-income countries confront unique difficulties due to a dearth of primary centres and delayed patient presentation. This results in patients that end up in tertiary hospitals being considered sicker and frailer. Although the vaccinated cohort in this study had advanced age, a higher CCI and a greater percentage of them developed severe ARDS. Nonetheless, the outcome was similar to nonvaccinated patients. Ferritin levels were also markedly elevated, in vaccinated patients.

A total of 133 patients were evaluated with severe to critical illness. The hospital mortality at our centre was 57.1%. It was found that despite advanced age and higher CCI in the vaccinated cohort, the outcome was comparable to nonvaccinated patients. Despite Pakistan having a broad-based population pyramid and one of the youngest populations in the world, our vaccinated cohort had a mean age of 60 years [15]. According to available data, this discrepancy emphasizes the positive impact of vaccination initiatives, as they protect against severe illness for younger individuals [16].

The vaccinated cohort comprised of older population; thus, a greater prevalence of concomitant chronic diseases was expected in them. This observation is supported by Motos et al. in their description of breakthrough infections in fully vaccinated patients, where they document a high rate of comorbidities among critically ill patients [17]. Brosh-Nissimov et al. chalked this up to decreased vaccine effectivity in such patients and an increased risk of exacerbation of underlying illness upon COVID-19 infection, thereby contributing to the severity of the disease [18].

Studies have analysed and demonstrated the beneficial impact of vaccination in the face of emerging virus variants and various vaccine types [19, 20]. However, the presence of a protective effect once critical illness has developed remains less clear. Furthermore, several risk factors for the development of severe disease have been identified in the literature, including age, gender and cardiovascular comorbidities [17]. In our cohort, the vaccinated patients were older and had higher CCI and a larger percentage developed severe ARDS upon ICU admission. They also exhibited marked elevations in ferritin levels. Moreover, they had a higher incidence of pulmonary artery hypertension, possibly due to their advanced age, comorbidities and functional limitations. Despite a worse clinical and laboratory picture and higher rates of complications including pulmonary artery hypertension in vaccinated patients, no significant difference between the length of ICU/hospital stay and mortality rates in comparison with nonvaccinated patients was found. This highlights the potential protective role of vaccines by triggering an immune response against the disease. The response is independent of vaccine type, even in the face of severe to critical COVID-19. The protective effect of the COVID-19 vaccine in elderly patients has been shown in a meta-analysis as well [21]. There have been similar outcomes reported by studies comparing outcomes among severely ill COVID-19 patients by vaccination status. Freund et al. echo these findings, reporting similar mortality rates in vaccinated and unvaccinated groups and demonstrating decreased oxygen needs among the vaccinated cohort [22]. It was also observed that in patients with severe ARDS, the vaccinated cohort had higher survival. Muthukrishnan et al. suggested decreased mortality among fully vaccinated patients who develop severe COVID-19 [23]. They also reported a positive association between rising inflammatory markers and the risk of mortality. The elevated ferritin levels and white blood cell count were significantly associated with mortality (*p* value: < 0.001).

Larger studies with representative samples will be required to analyse these associations and generate robust conclusions. Overall, we report a higher mortality rate than most published data, a finding reflective of the fact that very much critically ill patients were included in this study. There is a paucity of data on vaccine efficacy in LMIC settings. Most of the available data represents findings from high-income countries with excess access to vaccines. One study from Pakistan depicted better outcomes in vaccinated patients in comparison to nonvaccinated. In this cross-sectional study, all patients irrespective of disease severity were included. Thus, the majority of the population was below 40 years of age and 60 patients had critical COVID-19 [9]. This study presents data primarily with critical COVID-19 with a mean age of 60 years.

The patients had received a variety of vaccines, ranging from inactivated (Sinopharm, Sinovac, CanSinoBIO) to viral vector (Sputnik V, Oxford–AstraZeneca) to mRNA-based vaccines (Pfizer-BioNTech, Moderna) and evaluates their efficacy in a “real-world” setting. The conclusions drawn from this study can not only help direct further investigative efforts but also help guide policy on target populations for booster vaccination.

This study does, however, have some limitations. As a retrospective, observational study, the findings can infer association but cannot establish causality. Any complications developed after hospital discharge or subsequent readmission to a different centre cannot be commented upon. There is a need for large multicentre studies and pooled registry data analysis to generate robust conclusions to guide further practice.

## 5. Conclusion

To conclude, the study showed that vaccinated patients had comparable mortality to nonvaccinated patients despite being older and more ill upon admission to the hospital.

These findings highlight the significance of vaccination, especially for those who have a higher risk of severe or critical COVID-19.

## Figures and Tables

**Table 1:** Demographics and characteristics of hospital admission.

**Variables**	**Combined (N = 133)**	**Vaccination status**			**Hospital mortality**		
		**Yes (N = 64)**	**No (N = 69)**	**p value**	**Yes (N = 76)**	**No (N = 57)**	**p value**
Age (years)	60.0 ± 24.0	65.5 ± 21.3	50.0 ± 22.0	<0.001	65.0 ± 21.3	48.0 ± 24.0	<0.001
Age group				0.006			0.009
18–39	21 (15.8%)	7 (10.9%)	14 (20.3%)		8 (10.5%)	13 (22.8%)	
40–64	58 (43.6%)	22 (34.4%)	36 (52.2%)		29 (38.2%)	29 (50.9%)	
≥65	54 (40.6%)	35 (54.7%)	19 (27.5%)		39 (51.3%)	15 (26.3%)	
Gender				0.118			0.859
Female	56 (42.1%)	22 (34.4%)	34 (49.3%)		33 (43.4%)	23 (40.4%)	
Male	77 (57.9%)	42 (65.6%)	35 (50.7%)		43 (56.6%)	34 (59.6%)	
BMI	26.0 ± 5.40	26.0 ± 4.35	26.0 ± 5.40	0.900	25.2 ± 3.72	27.0 ± 5.80	0.007
Charlson Comorbidity Index	3.00 ± 4.00	3.00 ± 3.25	2.00 ± 5.00	0.021	3.00 ± 3.00	1.00 ± 4.00	0.003
Vaccinated patients	64 (48.1%)	—	—	—	36 (47.4%)	28 (49.1%)	0.841
Nonvaccinated patients	69 (51.9%)	—	—	—	40 (52.6%)	29 (50.9%)	0.841
Clinical characteristics on hospital admission							
Severe COVID-19	23 (17.3%)	7 (10.9%)	16 (23.2%)	0.061	10 (13.2%)	13 (22.8%)	0.144
Critical COVID-19	110 (82.7%)	57 (89.1%)	53 (76.8%)	0.061	66 (86.8%)	44 (77.2%)	0.144
Myocarditis	13 (9.77%)	3 (4.69%)	10 (14.5%)	0.057	9 (11.8%)	4 (7.02%)	0.352
Septic shock	41 (30.8%)	24 (37.5%)	17 (24.6%)	0.120	30 (39.5%)	11 (19.3%)	0.012
ARDS	120 (90.2%)	62 (96.9%)	58 (84.1%)	0.013	71 (93.4%)	49 (86.0%)	0.152
Laboratory parameters on hospital admission							
White blood cell count	14.2 ± 9.70	14.5 ± 8.95	13.5 ± 9.90	0.598	15.4 ± 11.0	12.5 ± 8.80	0.044
C-reactive protein	87.8 ± 119	105 ± 118	69.7 ± 122	0.770	83.5 ± 108	90.3 ± 130	0.750
Ferritin	949 ± 1140	1160 ± 917	651 ± 1260	0.219	1310 ± 1530	448 ± 957	<0.001
D-dimer	3.40 ± 7.20	3.55 ± 7.80	3.10 ± 5.60	0.959	4.15 ± 7.58	2.40 ± 6.30	0.154
PaO2/FiO2 ratio	142 ± 87.0	135 ± 79.0	145 ± 99.0	0.810	125 ± 70.0	170 ± 101	<0.001

Abbreviations: ARDS, acute respiratory distress syndrome; BMI, body mass index.

**Table 2:** Clinical characteristics during ICU stay and outcome.

**Variables**	**Combined (N = 133)**	**Vaccination status**			**Hospital mortality**		
		**Yes (N = 64)**	**No (N = 69)**	**p value**	**Yes (N = 76)**	**No (N = 57)**	**p value**
*Parameters during ICU stay*							
Infection during ICU stay	118 (88.7%)	55 (85.9%)	63 (91.3%)	0.327	70 (92.1%)	48 (84.2%)	0.156
MDR pathogen involved	68 (51.1%)	29 (45.3%)	39 (56.5%)	0.197	42 (55.3%)	26 (45.6%)	0.271
Remdesivir	89 (66.9%)	48 (75.0%)	41 (59.4%)	0.056	53 (69.7%)	36 (63.2%)	0.424
Tocilizumab	50 (37.6%)	30 (46.9%)	20 (29.0%)	0.033	34 (44.7%)	16 (28.1%)	0.050

*Organ support during ICU stay*							
Invasive ventilation	128 (96.2%)	60 (93.8%)	68 (98.6%)	0.147	74 (97.4%)	54 (94.7%)	0.423
Prone position	83 (62.4%)	41 (64.1%)	42 (60.9%)	0.704	49 (64.5%)	34 (59.6%)	0.569
Postextubation HFNC/NIV	31 (23.3%)	12 (18.8%)	19 (27.5%)	0.230	7 (9.21%)	24 (42.1%)	<0.001
Renal replacement therapy	31 (23.3%)	14 (21.9%)	17 (24.6%)	0.704	25 (32.9%)	6 (10.5%)	0.003
Cardiovascular support	89 (66.9%)	40 (62.5%)	49 (71.0%)	0.298	70 (92.1%)	19 (33.3%)	<0.001

*Complications during ICU stay*							
Total complications	107 (80.5%)	53 (82.8%)	54 (78.3%)	0.509	72 (94.7%)	35 (61.4%)	<0.001
Acute kidney injury	81 (60.9%)	41 (64.1%)	40 (58.0%)	0.472	59 (77.6%)	22 (38.6%)	<0.001
Cardiac arrhythmia requiring treatment	25 (18.8%)	14 (21.9%)	11 (15.9%)	0.379	20 (26.3%)	5 (8.77%)	0.010
Myocardial infarction	9 (6.77%)	1 (1.56%)	8 (11.6%)	0.021	7 (9.21%)	2 (3.51%)	0.194
Pneumothorax	19 (14.3%)	10 (15.6%)	9 (13.0%)	0.667	15 (19.7%)	4 (7.02%)	0.038
Pulmonary embolism	6 (4.51%)	2 (3.13%)	4 (5.80%)	0.459	4 (5.26%)	2 (3.51%)	0.631
Pulmonary artery hypertension	29 (21.8%)	19 (29.7%)	10 (14.5%)	0.034	23 (30.3%)	6 (10.5%)	0.006
Prolonged MV >20 days	20 (15.0%)	10 (15.6%)	10 (14.5%)	0.857	14 (18.4%)	6 (10.5%)	0.208
Reintubation	8 (6.02%)	3 (4.69%)	5 (7.25%)	0.535	4 (5.26%)	4 (7.02%)	0.675

*Outcome*							
ICU duration (days)	8.00 ± 8.00	8.50 ± 8.00	8.00 ± 7.00	0.995	10.0 ± 8.00	7.00 ± 5.00	0.003
Hospital duration (days)	14.0 ± 12.0	14.0 ± 9.75	14.0 ± 13.0	0.789	16.0 ± 14.0	14.0 ± 10.0	0.385
Days of invasive ventilation	8.00 ± 9.00	8.00 ± 9.00	8.00 ± 8.00	0.873	10.0 ± 10.0	6.00 ± 5.00	0.002
Hospital mortality	76 (57.1%)	36 (56.3%)	40 (58.0%)	0.841	—	—	—
ICU mortality	62 (46.6%)	30 (46.9%)	32 (46.4%)	0.952	—	—	—

Abbreviations: HFNC, high flow nasal cannula; ICU, intensive care unit; MDR, multidrug resistant; MV, mechanical ventilation; NIV, noninvasive ventilation.

**Table 3:** Factors associated with the vaccination status and hospital mortality.

**Factors**	**Adj. OR (95% CI)**	**p value**
*Vaccination status*		
Age	1.04 (1.01–1.071)	0.008
Male gender	2.35 (1.03–5.36)	0.043
Septic shock on ICU admission	2.28 (0.90–5.78)	0.082
C reactive protein	0.998 (0.994–1.002)	0.277
MDR pathogen involved	0.53 (0.24–1.19)	0.125
Tocilizumab	2.42 (0.98–5.95)	0.054
Cardiovascular support during ICU stay	0.27 (0.08–0.89)	0.031
Pulmonary artery hypertension	3.17 (1.11–9.03)	0.031
Hospital mortality	0.84 (0.29–2.39)	0.743

*Hospital mortality*		
Septic shock ICU admission	1.46 (0.55–3.84)	0.449
Ferritin	1.0002 (1–1.0005)	0.048
PaO2/FiO2 ratio on hospital admission	0.989 (0.983–0.995)	<0.001
Complications during ICU stay	8.81 (2.45–31.64)	<0.001
BMI	0.94 (0.86–1.034)	0.213
Vaccination status	0.64 (0.27–1.52)	0.312

Abbreviations: BMI, body mass index; MDR, multidrug resistant.

## Data Availability

Due to institutional policies and patient confidentiality regulations, the data from this study conducted at “The Aga Khan University Hospital” cannot be publicly shared or deposited in online repositories. However, interested researchers may contact the corresponding author to inquire about specific data queries or collaborations, while adhering to ethical and privacy considerations.
